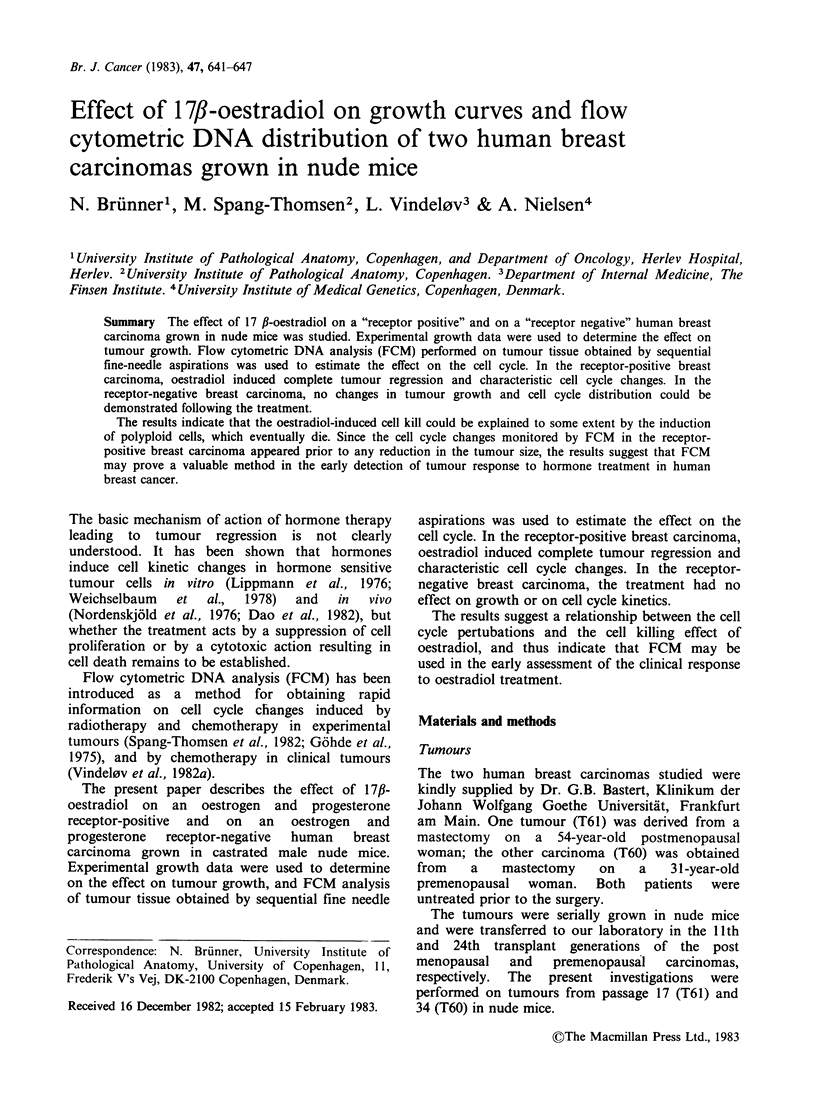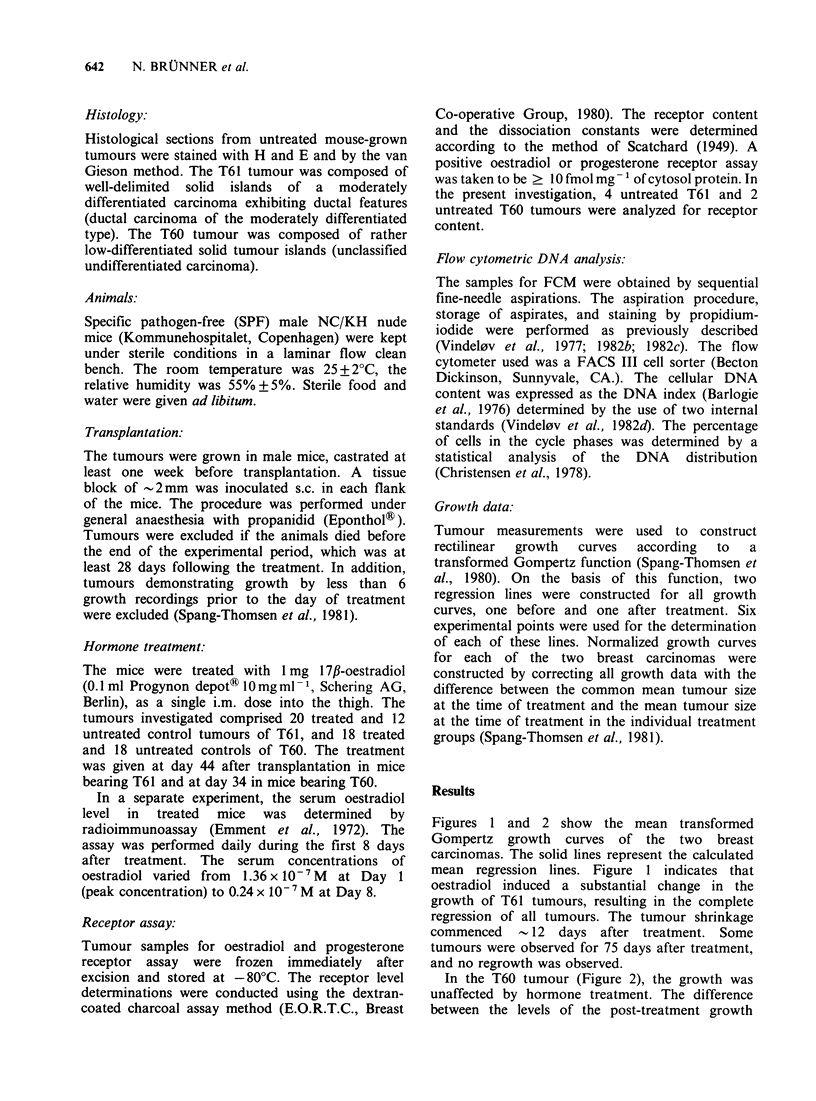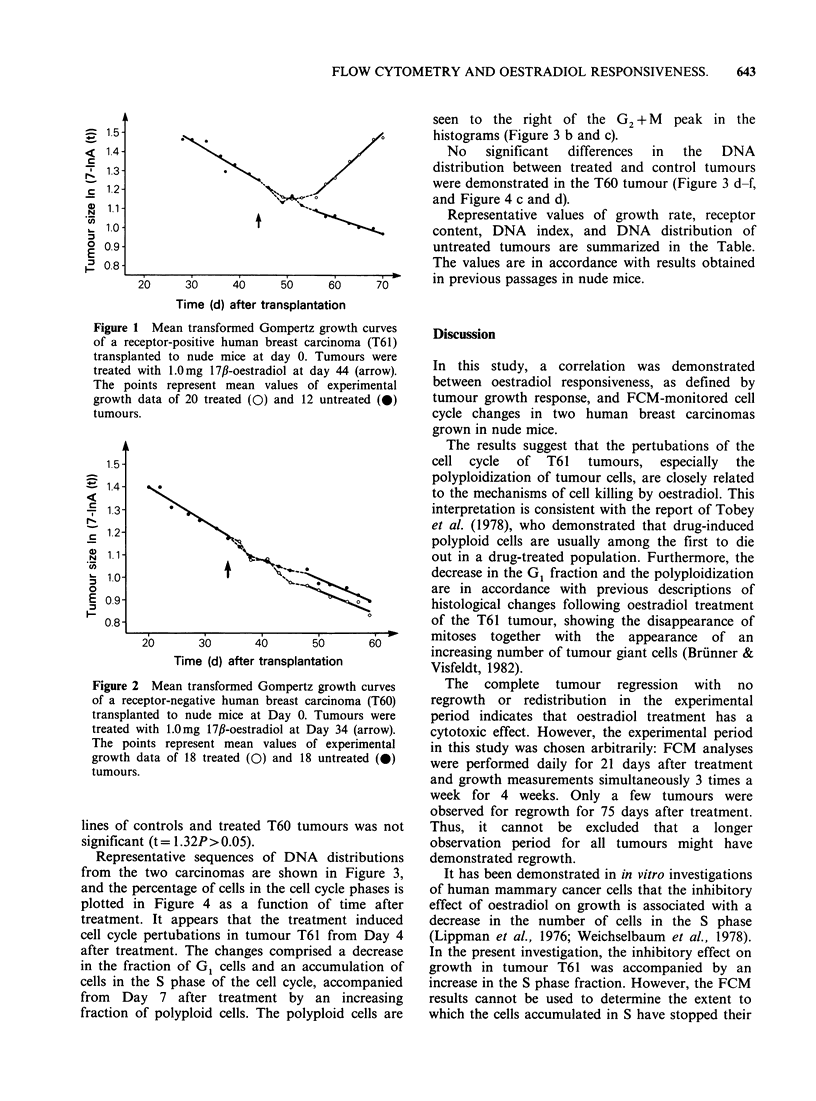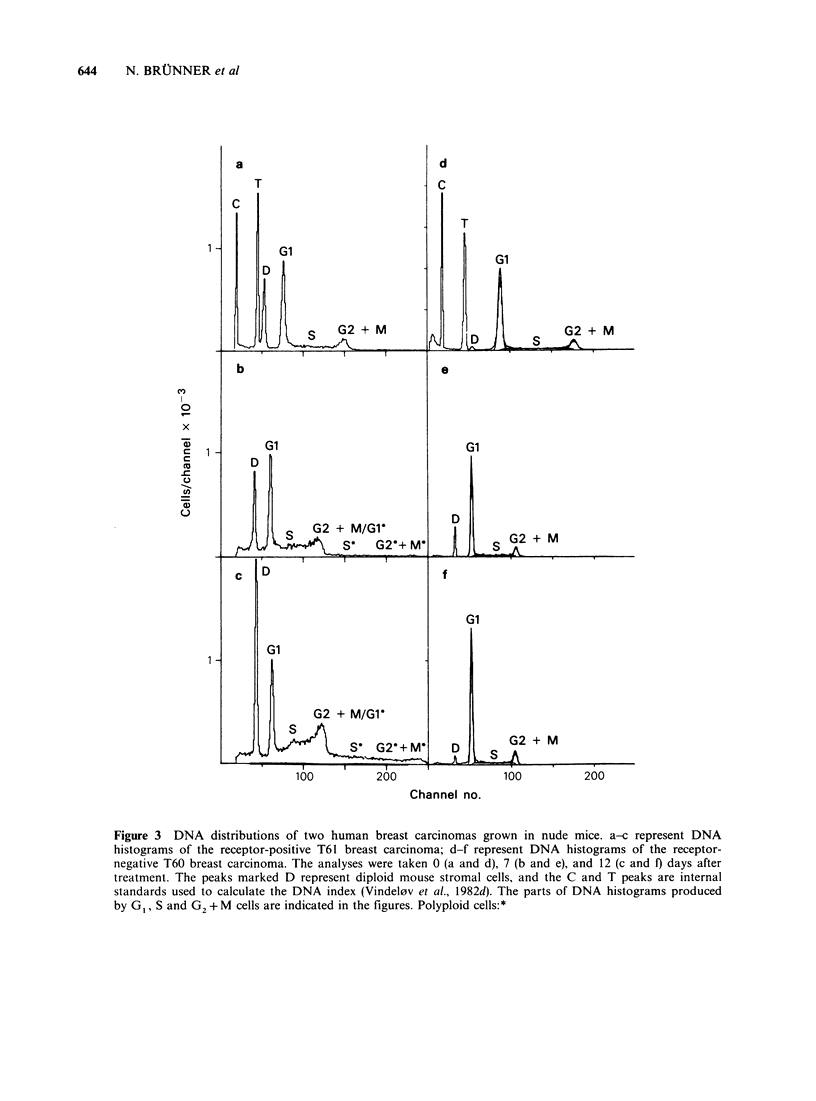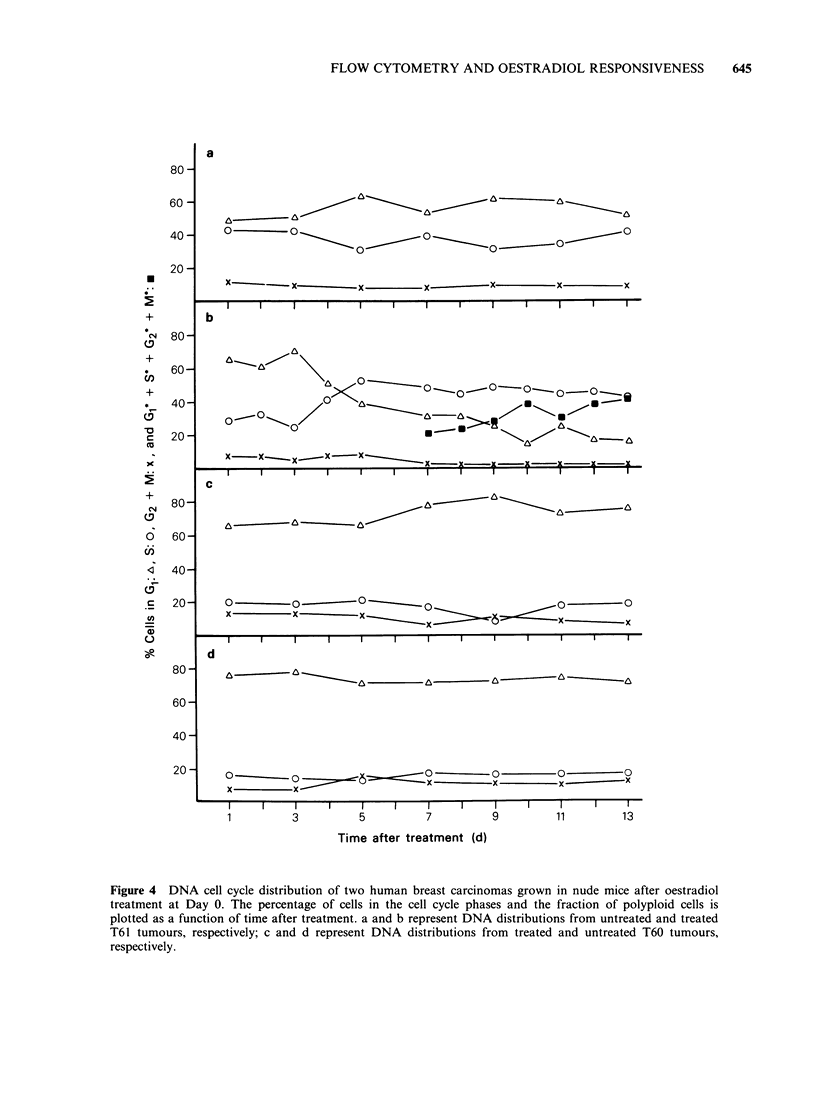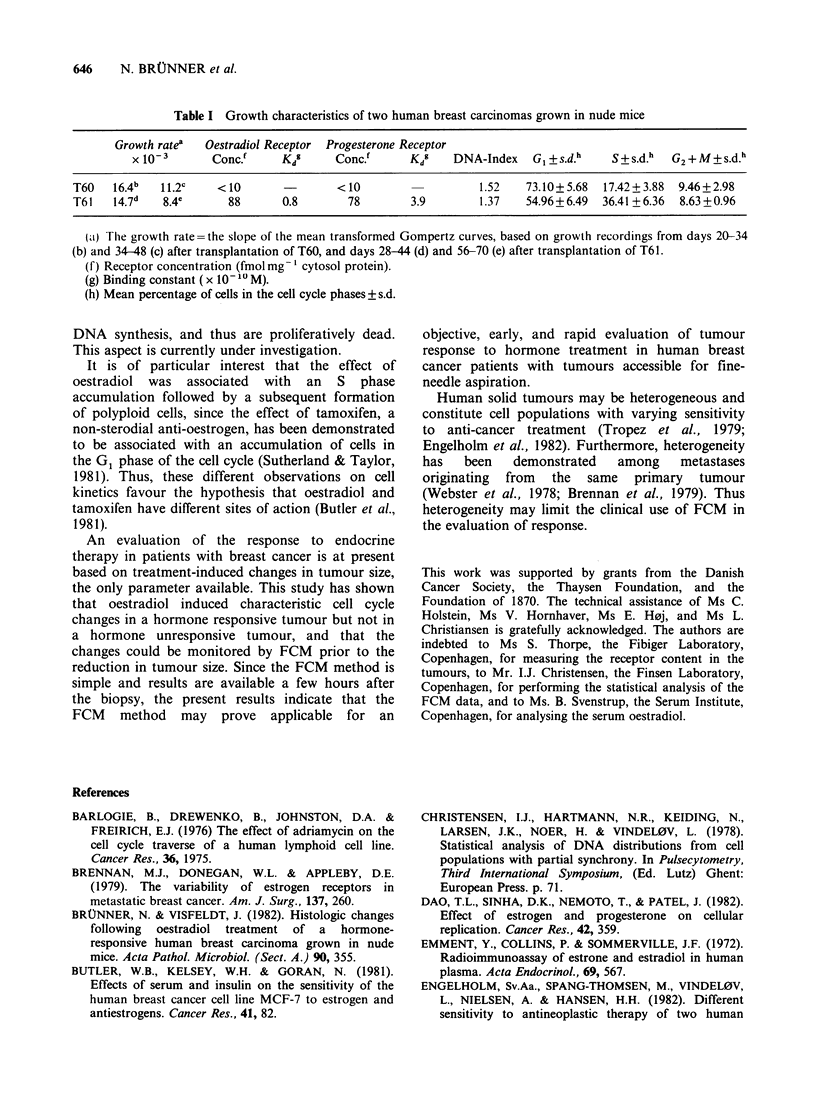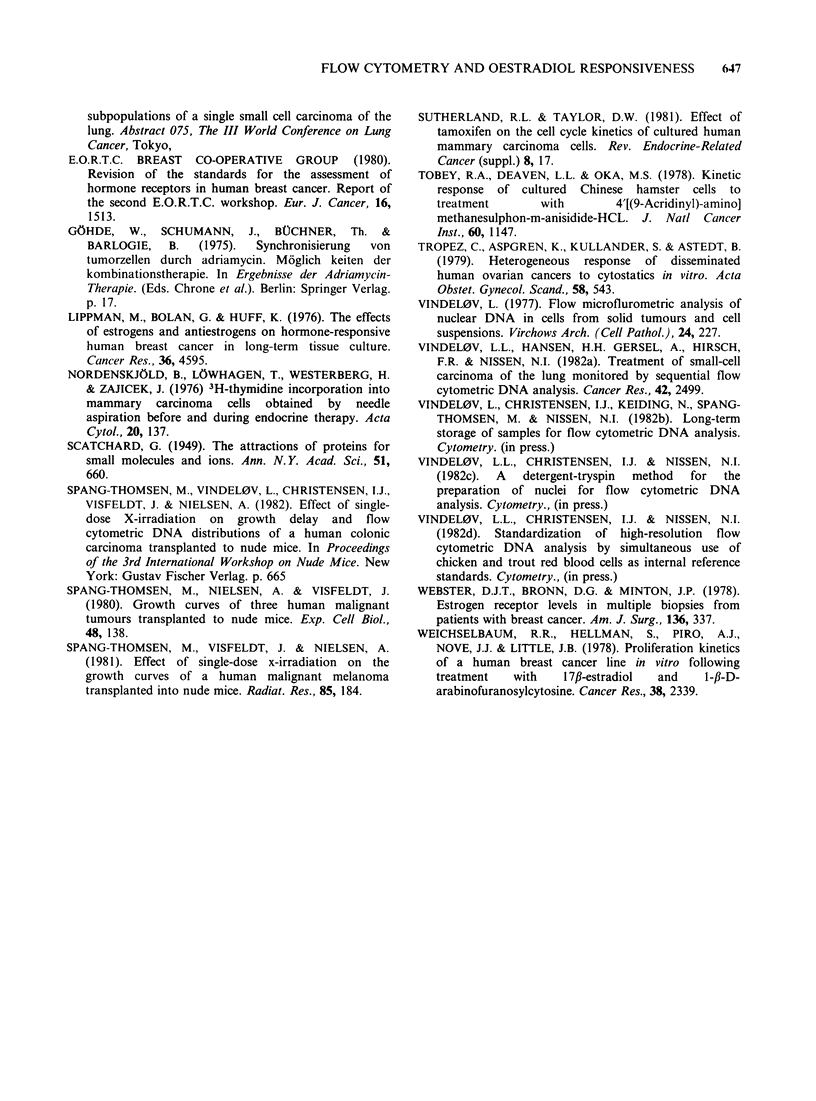# Effect of 17 beta-oestradiol on growth curves and flow cytometric DNA distribution of two human breast carcinomas grown in nude mice.

**DOI:** 10.1038/bjc.1983.102

**Published:** 1983-05

**Authors:** N. Brünner, M. Spang-Thomsen, L. Vindeløv, A. Nielsen

## Abstract

The effect of 17 beta-oestradiol on a "receptor positive" and on a "receptor negative" human breast carcinoma grown in nude mice was studied. Experimental growth data were used to determine the effect on tumour growth. Flow cytometric DNA analysis (FCM) performed on tumour tissue obtained by sequential fine-needle aspirations was used to estimate the effect on the cell cycle. In the receptor-positive breast carcinoma, oestradiol induced complete tumour regression and characteristic cell cycle changes. In the receptor-negative breast carcinoma, no changes in tumour growth and cell cycle distribution could be demonstrated following the treatment. The results indicate that the oestradiol-induced cell kill could be explained to some extent by the induction of polyploid cells, which eventually die. Since the cell cycle changes monitored by FCM in the receptor-positive breast carcinoma appeared prior to any reduction in the tumour size, the results suggest that FCM may prove a valuable method in the early detection of tumour response to hormone treatment in human breast cancer.


*Br. J. Cancer* (1983), **47**, 641–647

### Effect of 17β-oestradiol on growth curves and flow cytometric DNA distribution of two human breast carcinomas grown in nude mice

N. Brünner[1], M. Spang-Thomsen[2], L. Vindeløv[3] & A. Nielsen[4]

[1]*University Institute of Pathological Anatomy, Copenhagen, and Department of Oncology, Herlev Hospital, Herlev.* [2]*University Institute of Pathological Anatomy, Copenhagen.* [3]*Department of Internal Medicine, The Finsen Institute.* [4]*University Institute of Medical Genetics, Copenhagen, Denmark.*

**Summary** The effect of 17 β-oestradiol on a "receptor positive" and on a "receptor negative" human breast carcinoma grown in nude mice was studied. Experimental growth data were used to determine the effect on tumour growth. Flow cytometric DNA analysis (FCM) performed on tumour tissue obtained by sequential fine-needle aspirations was used to estimate the effect on the cell cycle. In the receptor-positive breast carcinoma, oestradiol induced complete tumour regression and characteristic cell cycle changes. In the receptor-negative breast carcinoma, no changes in tumour growth and cell cycle distribution could be demonstrated following the treatment.

The results indicate that the oestradiol-induced cell kill could be explained to some extent by the induction of polyploid cells, which eventually die. Since the cell cycle changes monitored by FCM in the receptor-positive breast carcinoma appeared prior to any reduction in the tumour size, the results suggest that FCM may prove a valuable method in the early detection of tumour response to hormone treatment in human breast cancer.

The basic mechanism of action of hormone therapy leading to tumour regression is not clearly understood. It has been shown that hormones induce cell kinetic changes in hormone sensitive tumour cells *in vitro* (Lippmann *et al.*, 1976; Weichselbaum *et al.*, 1978) and *in vivo* (Nordenskjöld *et al.*, 1976; Dao *et al.*, 1982), but whether the treatment acts by a suppression of cell proliferation or by a cytotoxic action resulting in cell death remains to be established.

Flow cytometric DNA analysis (FCM) has been introduced as a method for obtaining rapid information on cell cycle changes induced by radiotherapy and chemotherapy in experimental tumours (Spang-Thomsen *et al.*, 1982; Göhde *et al.*, 1975), and by chemotherapy in clinical tumours (Vindeløv *et al.*, 1982*a*).

The present paper describes the effect of 17β-oestradiol on an oestrogen and progesterone receptor-positive and on an oestrogen and progesterone receptor-negative human breast carcinoma grown in castrated male nude mice. Experimental growth data were used to determine on the effect on tumour growth, and FCM analysis of tumour tissue obtained by sequential fine needle aspirations was used to estimate the effect on the cell cycle. In the receptor-positive breast carcinoma, oestradiol induced complete tumour regression and characteristic cell cycle changes. In the receptor-negative breast carcinoma, the treatment had no effect on growth or on cell cycle kinetics.

The results suggest a relationship between the cell cycle pertubations and the cell killing effect of oestradiol, and thus indicate that FCM may be used in the early assessment of the clinical response to oestradiol treatment.

Correspondence: N. Brünner, University Institute of Pathological Anatomy, University of Copenhagen, 11, Frederik V's Vej, DK-2100 Copenhagen, Denmark.

Received 16 December 1982; accepted 15 February 1983.

### Materials and methods

### *Tumours*

The two human breast carcinomas studied were kindly supplied by Dr. G.B. Bastert, Klinikum der Johann Wolfgang Goethe Universität, Frankfurt am Main. One tumour (T61) was derived from a mastectomy on a 54-year-old postmenopausal woman; the other carcinoma (T60) was obtained from a mastectomy on a 31-year-old premenopausal woman. Both patients were untreated prior to the surgery.

The tumours were serially grown in nude mice and were transferred to our laboratory in the 11th and 24th transplant generations of the post menopausal and premenopausal carcinomas, respectively. The present investigations were performed on tumours from passage 17 (T61) and 34 (T60) in nude mice.

©The Macmillan Press Ltd., 1983

*Histology:*

Histological sections from untreated mouse-grown tumours were stained with H and E and by the van Gieson method. The T61 tumour was composed of well-delimited solid islands of a moderately differentiated carcinoma exhibiting ductal features (ductal carcinoma of the moderately differentiated type). The T60 tumour was composed of rather low-differentiated solid tumour islands (unclassified undifferentiated carcinoma).

*Animals:*

Specific pathogen-free (SPF) male NC/KH nude mice (Kommunehospitalet, Copenhagen) were kept under sterile conditions in a laminar flow clean bench. The room temperature was 25±2°C, the relative humidity was 55%±5%. Sterile food and water were given *ad libitum*.

*Transplantation:*

The tumours were grown in male mice, castrated at least one week before transplantation. A tissue block of ~2mm was inoculated s.c. in each flank of the mice. The procedure was performed under general anaesthesia with propanidid (Eponthol®). Tumours were excluded if the animals died before the end of the experimental period, which was at least 28 days following the treatment. In addition, tumours demonstrating growth by less than 6 growth recordings prior to the day of treatment were excluded (Spang-Thomsen *et al.*, 1981).

*Hormone treatment:*

The mice were treated with 1 mg 17β-oestradiol (0.1 ml Progynon depot® 10 mg ml$^{-1}$, Schering AG, Berlin), as a single i.m. dose into the thigh. The tumours investigated comprised 20 treated and 12 untreated control tumours of T61, and 18 treated and 18 untreated controls of T60. The treatment was given at day 44 after transplantation in mice bearing T61 and at day 34 in mice bearing T60.

In a separate experiment, the serum oestradiol level in treated mice was determined by radioimmunoassay (Emment *et al.*, 1972). The assay was performed daily during the first 8 days after treatment. The serum concentrations of oestradiol varied from $1.36 \times 10^{-7}$ M at Day 1 (peak concentration) to $0.24 \times 10^{-7}$ M at Day 8.

*Receptor assay:*

Tumour samples for oestradiol and progesterone receptor assay were frozen immediately after excision and stored at −80°C. The receptor level determinations were conducted using the dextran-coated charcoal assay method (E.O.R.T.C., Breast Co-operative Group, 1980). The receptor content and the dissociation constants were determined according to the method of Scatchard (1949). A positive oestradiol or progesterone receptor assay was taken to be ≥ 10 fmol mg$^{-1}$ of cytosol protein. In the present investigation, 4 untreated T61 and 2 untreated T60 tumours were analyzed for receptor content.

*Flow cytometric DNA analysis:*

The samples for FCM were obtained by sequential fine-needle aspirations. The aspiration procedure, storage of aspirates, and staining by propidium-iodide were performed as previously described (Vindeløv *et al.*, 1977; 1982*b*; 1982*c*). The flow cytometer used was a FACS III cell sorter (Becton Dickinson, Sunnyvale, CA.). The cellular DNA content was expressed as the DNA index (Barlogie *et al.*, 1976) determined by the use of two internal standards (Vindeløv *et al.*, 1982*d*). The percentage of cells in the cycle phases was determined by a statistical analysis of the DNA distribution (Christensen *et al.*, 1978).

*Growth data:*

Tumour measurements were used to construct rectilinear growth curves according to a transformed Gompertz function (Spang-Thomsen *et al.*, 1980). On the basis of this function, two regression lines were constructed for all growth curves, one before and one after treatment. Six experimental points were used for the determination of each of these lines. Normalized growth curves for each of the two breast carcinomas were constructed by correcting all growth data with the difference between the common mean tumour size at the time of treatment and the mean tumour size at the time of treatment in the individual treatment groups (Spang-Thomsen *et al.*, 1981).

### Results

Figures 1 and 2 show the mean transformed Gompertz growth curves of the two breast carcinomas. The solid lines represent the calculated mean regression lines. Figure 1 indicates that oestradiol induced a substantial change in the growth of T61 tumours, resulting in the complete regression of all tumours. The tumour shrinkage commenced ~12 days after treatment. Some tumours were observed for 75 days after treatment, and no regrowth was observed.

In the T60 tumour (Figure 2), the growth was unaffected by hormone treatment. The difference between the levels of the post-treatment growth

**Figure 1** Mean transformed Gompertz growth curves of a receptor-positive human breast carcinoma (T61) transplanted to nude mice at day 0. Tumours were treated with 1.0 mg 17β-oestradiol at day 44 (arrow). The points represent mean values of experimental growth data of 20 treated (○) and 12 untreated (●) tumours.

**Figure 2** Mean transformed Gompertz growth curves of a receptor-negative human breast carcinoma (T60) transplanted to nude mice at Day 0. Tumours were treated with 1.0 mg 17β-oestradiol at Day 34 (arrow). The points represent mean values of experimental growth data of 18 treated (○) and 18 untreated (●) tumours.

lines of controls and treated T60 tumours was not significant ($t = 1.32 P > 0.05$).

Representative sequences of DNA distributions from the two carcinomas are shown in Figure 3, and the percentage of cells in the cell cycle phases is plotted in Figure 4 as a function of time after treatment. It appears that the treatment induced cell cycle pertubations in tumour T61 from Day 4 after treatment. The changes comprised a decrease in the fraction of $G_1$ cells and an accumulation of cells in the S phase of the cell cycle, accompanied from Day 7 after treatment by an increasing fraction of polyploid cells. The polyploid cells are seen to the right of the $G_2+M$ peak in the histograms (Figure 3 b and c).

No significant differences in the DNA distribution between treated and control tumours were demonstrated in the T60 tumour (Figure 3 d–f, and Figure 4 c and d).

Representative values of growth rate, receptor content, DNA index, and DNA distribution of untreated tumours are summarized in the Table. The values are in accordance with results obtained in previous passages in nude mice.

### Discussion

In this study, a correlation was demonstrated between oestradiol responsiveness, as defined by tumour growth response, and FCM-monitored cell cycle changes in two human breast carcinomas grown in nude mice.

The results suggest that the pertubations of the cell cycle of T61 tumours, especially the polyploidization of tumour cells, are closely related to the mechanisms of cell killing by oestradiol. This interpretation is consistent with the report of Tobey *et al.* (1978), who demonstrated that drug-induced polyploid cells are usually among the first to die out in a drug-treated population. Furthermore, the decrease in the $G_1$ fraction and the polyploidization are in accordance with previous descriptions of histological changes following oestradiol treatment of the T61 tumour, showing the disappearance of mitoses together with the appearance of an increasing number of tumour giant cells (Brünner & Visfeldt, 1982).

The complete tumour regression with no regrowth or redistribution in the experimental period indicates that oestradiol treatment has a cytotoxic effect. However, the experimental period in this study was chosen arbitrarily: FCM analyses were performed daily for 21 days after treatment and growth measurements simultaneously 3 times a week for 4 weeks. Only a few tumours were observed for regrowth for 75 days after treatment. Thus, it cannot be excluded that a longer observation period for all tumours might have demonstrated regrowth.

It has been demonstrated in *in vitro* investigations of human mammary cancer cells that the inhibitory effect of oestradiol on growth is associated with a decrease in the number of cells in the S phase (Lippman *et al.*, 1976; Weichselbaum *et al.*, 1978). In the present investigation, the inhibitory effect on growth in tumour T61 was accompanied by an increase in the S phase fraction. However, the FCM results cannot be used to determine the extent to which the cells accumulated in S have stopped their

**Figure 3** DNA distributions of two human breast carcinomas grown in nude mice. a–c represent DNA histograms of the receptor-positive T61 breast carcinoma; d–f represent DNA histograms of the receptor-negative T60 breast carcinoma. The analyses were taken 0 (a and d), 7 (b and e), and 12 (c and f) days after treatment. The peaks marked D represent diploid mouse stromal cells, and the C and T peaks are internal standards used to calculate the DNA index (Vindeløv *et al.*, 1982*d*). The parts of DNA histograms produced by $G_1$, S and $G_2$+M cells are indicated in the figures. Polyploid cells:*

**Figure 4** DNA cell cycle distribution of two human breast carcinomas grown in nude mice after oestradiol treatment at Day 0. The percentage of cells in the cell cycle phases and the fraction of polyploid cells is plotted as a function of time after treatment. a and b represent DNA distributions from untreated and treated T61 tumours, respectively; c and d represent DNA distributions from treated and untreated T60 tumours, respectively.

**Table I** Growth characteristics of two human breast carcinomas grown in nude mice

| | *Growth rate*[a] $\times 10^{-3}$ | | *Oestradiol Receptor* Conc.[f] | $K_d$[g] | *Progesterone Receptor* Conc.[f] | $K_d$[g] | DNA-Index | $G_1 \pm s.d.$[h] | $S \pm$ s.d.[h] | $G_2 + M \pm$ s.d.[h] |
|---|---|---|---|---|---|---|---|---|---|---|
| T60 | 16.4[b] | 11.2[c] | <10 | — | <10 | — | 1.52 | 73.10±5.68 | 17.42±3.88 | 9.46±2.98 |
| T61 | 14.7[d] | 8.4[e] | 88 | 0.8 | 78 | 3.9 | 1.37 | 54.96±6.49 | 36.41±6.36 | 8.63±0.96 |

(a) The growth rate = the slope of the mean transformed Gompertz curves, based on growth recordings from days 20–34 (b) and 34–48 (c) after transplantation of T60, and days 28–44 (d) and 56–70 (e) after transplantation of T61.
(f) Receptor concentration (fmol mg$^{-1}$ cytosol protein).
(g) Binding constant ($\times 10^{-10}$ M).
(h) Mean percentage of cells in the cell cycle phases ± s.d.

DNA synthesis, and thus are proliferatively dead. This aspect is currently under investigation.

It is of particular interest that the effect of oestradiol was associated with an S phase accumulation followed by a subsequent formation of polyploid cells, since the effect of tamoxifen, a non-sterodial anti-oestrogen, has been demonstrated to be associated with an accumulation of cells in the $G_1$ phase of the cell cycle (Sutherland & Taylor, 1981). Thus, these different observations on cell kinetics favour the hypothesis that oestradiol and tamoxifen have different sites of action (Butler *et al.*, 1981).

An evaluation of the response to endocrine therapy in patients with breast cancer is at present based on treatment-induced changes in tumour size, the only parameter available. This study has shown that oestradiol induced characteristic cell cycle changes in a hormone responsive tumour but not in a hormone unresponsive tumour, and that the changes could be monitored by FCM prior to the reduction in tumour size. Since the FCM method is simple and results are available a few hours after the biopsy, the present results indicate that the FCM method may prove applicable for an objective, early, and rapid evaluation of tumour response to hormone treatment in human breast cancer patients with tumours accessible for fine-needle aspiration.

Human solid tumours may be heterogeneous and constitute cell populations with varying sensitivity to anti-cancer treatment (Tropez *et al.*, 1979; Engelholm *et al.*, 1982). Furthermore, heterogeneity has been demonstrated among metastases originating from the same primary tumour (Webster *et al.*, 1978; Brennan *et al.*, 1979). Thus heterogeneity may limit the clinical use of FCM in the evaluation of response.

This work was supported by grants from the Danish Cancer Society, the Thaysen Foundation, and the Foundation of 1870. The technical assistance of Ms C. Holstein, Ms V. Hornhaver, Ms E. Høj, and Ms L. Christiansen is gratefully acknowledged. The authors are indebted to Ms S. Thorpe, the Fibiger Laboratory, Copenhagen, for measuring the receptor content in the tumours, to Mr. I.J. Christensen, the Finsen Laboratory, Copenhagen, for performing the statistical analysis of the FCM data, and to Ms. B. Svenstrup, the Serum Institute, Copenhagen, for analysing the serum oestradiol.

### References

BARLOGIE, B., DREWENKO, B., JOHNSTON, D.A. & FREIRICH, E.J. (1976) The effect of adriamycin on the cell cycle traverse of a human lymphoid cell line. *Cancer Res.*, **36**, 1975.

BRENNAN, M.J., DONEGAN, W.L. & APPLEBY, D.E. (1979). The variability of estrogen receptors in metastatic breast cancer. *Am. J. Surg.*, **137**, 260.

BRÜNNER, N. & VISFELDT, J. (1982). Histologic changes following oestradiol treatment of a hormone-responsive human breast carcinoma grown in nude mice. *Acta Pathol. Microbiol. (Sect. A.)* **90**, 355.

BUTLER, W.B., KELSEY, W.H. & GORAN, N. (1981). Effects of serum and insulin on the sensitivity of the human breast cancer cell line MCF-7 to estrogen and antiestrogens. *Cancer Res.*, **41**, 82.

CHRISTENSEN, I.J., HARTMANN, N.R., KEIDING, N., LARSEN, J.K., NOER, H. & VINDELØV, L. (1978). Statistical analysis of DNA distributions from cell populations with partial synchrony. In *Pulsecytometry, Third International Symposium*, (Ed. Lutz) Ghent: European Press. p. 71.

DAO, T.L., SINHA, D.K., NEMOTO, T., & PATEL, J. (1982). Effect of estrogen and progesterone on cellular replication. *Cancer Res.*, **42**, 359.

EMMENT, Y., COLLINS, P. & SOMMERVILLE, J.F. (1972). Radioimmunoassay of estrone and estradiol in human plasma. *Acta Endocrinol.*, **69**, 567.

ENGELHOLM, Sv.Aa., SPANG-THOMSEN, M., VINDELØV, L., NIELSEN, A. & HANSEN, H.H. (1982). Different sensitivity to antineoplastic therapy of two human subpopulations of a single small cell carcinoma of the lung. *Abstract 075, The III World Conference on Lung Cancer*, Tokyo,

E.O.R.T.C. BREAST CO-OPERATIVE GROUP (1980). Revision of the standards for the assessment of hormone receptors in human breast cancer. Report of the second E.O.R.T.C. workshop. *Eur. J. Cancer*, **16**, 1513.

GÖHDE, W., SCHUMANN, J., BÜCHNER, Th. & BARLOGIE, B. (1975). Synchronisierung von tumorzellen durch adriamycin. Möglich keiten der kombinationstherapie. In *Ergebnisse der Adriamycin-Therapie*. (Eds. Chrone *et al.*). Berlin: Springer Verlag. p. 17.

LIPPMAN, M., BOLAN, G. & HUFF, K. (1976). The effects of estrogens and antiestrogens on hormone-responsive human breast cancer in long-term tissue culture. *Cancer Res.*, **36**, 4595.

NORDENSKJÖLD, B., LÖWHAGEN, T., WESTERBERG, H. & ZAJICEK, J. (1976) $^3$H-thymidine incorporation into mammary carcinoma cells obtained by needle aspiration before and during endocrine therapy. *Acta Cytol.*, **20**, 137.

SCATCHARD, G. (1949). The attractions of proteins for small molecules and ions. *Ann. N.Y. Acad. Sci.*, **51**, 660.

SPANG-THOMSEN, M., VINDELØV, L., CHRISTENSEN, I.J., VISFELDT, J. & NIELSEN, A. (1982). Effect of single-dose X-irradiation on growth delay and flow cytometric DNA distributions of a human colonic carcinoma transplanted to nude mice. In *Proceedings of the 3rd International Workshop on Nude Mice*. New York: Gustav Fischer Verlag. p. 665

SPANG-THOMSEN, M., NIELSEN, A. & VISFELDT, J. (1980). Growth curves of three human malignant tumours transplanted to nude mice. *Exp. Cell Biol.*, **48**, 138.

SPANG-THOMSEN, M., VISFELDT, J. & NIELSEN, A. (1981). Effect of single-dose x-irradiation on the growth curves of a human malignant melanoma transplanted into nude mice. *Radiat. Res.*, **85**, 184.

SUTHERLAND, R.L. & TAYLOR, D.W. (1981). Effect of tamoxifen on the cell cycle kinetics of cultured human mammary carcinoma cells. *Rev. Endocrine-Related Cancer* (suppl.) **8**, 17.

TOBEY, R.A., DEAVEN, L.L. & OKA, M.S. (1978). Kinetic response of cultured Chinese hamster cells to treatment with 4′[(9-Acridinyl)-amino] methanesulphon-m-anisidide-HCL. *J. Natl Cancer Inst.*, **60**, 1147.

TROPEZ, C., ASPGREN, K., KULLANDER, S. & ASTEDT, B. (1979). Heterogeneous response of disseminated human ovarian cancers to cytostatics *in vitro*. *Acta Obstet. Gynecol. Scand.*, **58**, 543.

VINDELØV, L. (1977). Flow microflurometric analysis of nuclear DNA in cells from solid tumours and cell suspensions. *Virchows Arch. (Cell Pathol.)*, **24**, 227.

VINDELØV, L.L., HANSEN, H.H. GERSEL, A., HIRSCH, F.R. & NISSEN, N.I. (1982a). Treatment of small-cell carcinoma of the lung monitored by sequential flow cytometric DNA analysis. *Cancer Res.*, **42**, 2499.

VINDELØV, L., CHRISTENSEN, I.J., KEIDING, N., SPANG-THOMSEN, M. & NISSEN, N.I. (1982b). Long-term storage of samples for flow cytometric DNA analysis. *Cytometry*. (in press.)

VINDELØV, L.L., CHRISTENSEN, I.J. & NISSEN, N.I. (1982c). A detergent-tryspin method for the preparation of nuclei for flow cytometric DNA analysis. *Cytometry.*, (in press.)

VINDELØV, L.L., CHRISTENSEN, I.J. & NISSEN, N.I. (1982d). Standardization of high-resolution flow cytometric DNA analysis by simultaneous use of chicken and trout red blood cells as internal reference standards. *Cytometry.*, (in press.)

WEBSTER, D.J.T., BRONN, D.G. & MINTON, J.P. (1978). Estrogen receptor levels in multiple biopsies from patients with breast cancer. *Am. J. Surg.*, **136**, 337.

WEICHSELBAUM, R.R., HELLMAN, S., PIRO, A.J., NOVE, J.J. & LITTLE, J.B. (1978). Proliferation kinetics of a human breast cancer line *in vitro* following treatment with 17$\beta$-estradiol and 1-$\beta$-D-arabinofuranosylcytosine. *Cancer Res.*, **38**, 2339.